# Risk factors for HCV transmission in HIV-positive men who have sex with men in México

**DOI:** 10.1371/journal.pone.0269977

**Published:** 2022-07-15

**Authors:** José Antonio Mata-Marín, Armando Abraham de Pablos-Leal, Stefan Mauss, Carla Ileana Arroyo-Anduiza, Mara Soraya Rodríguez-Evaristo, Luis Antonio Uribe-Noguéz, María de los Ángeles Berrospe-Silva, Juan Carlos Lara-Castañeda, Edgar Pérez-Barragán, Jesús Gaytán-Martínez

**Affiliations:** 1 Infectious Diseases Department, Hospital de Infectología "La Raza" National Medical Center, Instituto Mexicano del Seguro Social, Mexico City, México; 2 Center for HIV and Hepatogastroenterology, Duesseldorf, Germany; 3 Banco Central de Sangre "La Raza" National Medical Center, Instituto Mexicano del Seguro Social, Mexico City, México; 4 Departamento de Ciencias de la Salud, Campus Cajeme, Universidad de Sonora, Ciudad Obregón, Sonora, México; 5 Laboratorio Central de Epidemiología, "La Raza" National Medical Center, Instituto Mexicano del Seguro Social, Mexico City, México; 6 Infectious Diseases Department, Unidad de Infectología “Juan I. Menchaca”, Instituto Mexicano del Seguro Social, Guadalajara, Jalisco, México; 7 Infectious Diseases Department, Hospital General Regional No. 66 “Dr. Héctor J. Moyetón Contreras”, Instituto Mexicano del Seguro Social, Ciudad Juárez, Chihuahua, México; 8 Infectious Diseases Department, Hospital General de Zona No. 48 “San Pedro Xalpa”, Instituto Mexicano del Seguro Social, Mexico City, México; George Washington University, UNITED STATES

## Abstract

**Purpose:**

In the last two decades transmission of hepatitis C virus (HCV) in HIV positive men who have sex with men (MSM) has been reported globally. Chemsex and specific sexual practices have been identified as risk factors. Our study aimed to identify risk factors for HCV transmission in MSM living with HIV attending in Mexico.

**Methods:**

We conducted a case-control study from April to December 2019 at the Hospital de Infectología “La Raza” National Medical Center, in Mexico City. A case was defined as an HIV-infected MSM with positive HCV-antibody test. For each case, 3 controls were included, defined as HIV infected MSM with negative HCV-antibody test. A self-questionnaire covering sexual practices and other risk factors for HCV transmission was applied. Bivariate analysis was performed to obtain odds ratio (OR) using Chi-square test. Independent risk factors were identified in a subsequent analysis performing a logistic regression model.

**Results:**

A total of 324 patients participated in the study, 81 cases and 243 controls. Median age was 30.5 years (IQR: 18–52) and 28.8 years (IQR: 21–45) in the case and control group, respectively. Most prevalent HCV genotype was 1a (79%). In the logistic regression model, sharing straw during cocaine inhalation (OR: 9.03; 95% CI; 1.35–13.52; P = 0.003), sharing sex toys (OR: 17.53, 95% CI; 6.85–44.86; P = 0.002), and ethyl chloride use for chemsex (OR: 2.26; 95% CI; 1.29–5.56; P = 0.037) were significant risk factors for HCV infection.

**Conclusion:**

This study identifies risk factors for HCV transmission in Mexico in HIV positive MSM in congruence with the findings of many studies performed worldwide. This is the first study that indicates a possible association between ethyl chloride use in chemsex and HCV infection. Assessment of local populations for risk factors for HCV transmission may help to develop specifically targeted behavioral interventions to reduce HCV transmission.

## Introduction

Globally more than 71.1 million people are infected with hepatitis C virus (HCV) [1], and 38 million people with human immunodeficiency virus (HIV), Mexico however, has a low HIV prevalence in adults (0.54%) [2, 3]. HCV is primarily transmitted through percutaneous exposure to blood, owing to medical procedures or sharing contaminated devices for injection drug use (IDU) [4].

Over the last 2 decades, effective harm reduction practices have contributed to the minimization of HIV transmission in IDU, thereby concomitantly reducing exposure to HCV. However, HCV infection in non-IDU men who have sex with men (MSM), has been on the rise at the same time and gained increasing attention [5].

Sexual transmission of HCV has emerged as a risk factor for HCV infection since 2000 in MSM particularly in HIV-positive individuals [6]. Certain sexual practices involving trauma of the rectal mucosa have been discussed as relevant risk factors for transmission among MSM. A German study involving 34 cases and 67 controls identified frequent rectal trauma with bleeding, receptive fisting without gloves, group sex, use of sex toys and nasally administered drugs [7, 8]. Another risk factor identified in the prospectively recruited MACS cohort (Multicenter AIDS Cohort Study) was an association between HCV seropositivity and enema use before receptive anal intercourse [9]. Mucosal ulcerative sexually transmitted infections (STIs) such as syphilis may also facilitate HCV acquisition through disrupting mucosal integrity [10].

There is also evidence that “chemsex”, defined as the voluntary intake of certain psychoactive and non-psychoactive drugs in the context of sex parties and sexual intercourses with the intention of facilitating and/or enhancing the sexual encounter, mostly among men who have sex with other men, is associated with HCV sexual transmission [11–15].

The findings show the importance of screening HIV-infected MSM for HCV, particularly those engaged in high-risk sexual behavior. In addition, understanding specific risk factors for HCV transmission, which may vary in the local MSM population, is important for behavioral interventions.

The aim of this study was to identify the risk factors for HCV infection in HIV positive MSM in Mexico City.

## Methods

### Design

A case-control study 1:3 was conducted from April to December 2019, in patients treated at the Hospital de Infectología “La Raza” National Medical Center, Mexico City.

The HIV clinic is a third level reference center for people with social security coverage. Only male patients attend and patients are frequently referred for initiation of antiretroviral therapy and relocated to their local clinics after completing the first treatment period.

The study was approved by the local committee for health research 0350 at the Hospital de Infectología “La Raza” National Medical Center, with registration number R-2810-003-002. Written informed consent was obtained from all participants before answering a risk factors questionnaire.

### Study population

Study subjects were HIV-infected MSM ≥18 years old. For this study, a case-patient was defined as an HIV-infected MSM evaluated at National Medical Center “La Raza”, who had a positive HCV-antibody test result. For each case-patient, 3 controls were included, defined as HIV infected MSM who had a negative HCV-antibody test. We retested all cases with HCV antibody test (ELISA) and HCV RNA.

### Measurements

Information was obtained in an anonymous 44-item self-questionnaire covering sexual behavior, use of illicit or recreational drugs and diagnoses of STIs. We used a modified version of the questionnaire of Zuure *et al*., and added some risk factors like chemsex and drugs that are common in Mexico for recreational drug use [16, 17] S1 Appendix. For example, ethyl chloride can be easily accessed in drug stores and is therefore frequently used in Mexico. In addition, clinical data were collected as following: previous serological diagnosis of syphilis, diagnosis of urethritis, genital ulcers, proctitis, HBV infection and antiretroviral therapy (ART).

The laboratory results performed in cases and controls were CD4+ cell count, HIV RNA and in HCV cases, HCV RNA and HCV genotype. We used Architect anti‐HCV assay (Abbott, Wiesbaden, Germany) for HCV antibody testing and Abbott RealTime HCV (Abbott Molecular Inc., Des Plaines, IL, USA) for quantitative HCV RNA.

### Statistical analysis

The sample size was calculated with an 95% confidence level and an 80% of power, using a formula for unmatched Case-Control studies. In the case of non-normal distributions, we used the Kolmogorov Smirnov test. Descriptive results were summarized using median and interquartile ranges (IQR).

Bivariate analysis was performed to evaluate the risk factors for HCV transmission and to obtain the odds ratio (OR) using the Chi-square test and Fisher’s exact test. Independent risk factors associated with HCV transmission were identified in the logistic regression analysis (calculated with 95% confidence intervals) which included the significant variables (p<0.05) from the bivariate analysis. All analyses were conducted using SPSS software (version 22; SPSS IBM Corp., Armonk, NJ, USA).

## Results

Of the 337 patients invited to participate in the study, 81 cases and 243 controls agreed to participate and 13 declined the invitation. The median age was 30.5 years (IQR: 18–52) for cases and 28.8 years (IQR: 21–45) for controls. The median CD4+ cell counts of cases were 498 cells/mm^3^ and 324 cells/mm^3^ in the control group. Of the 81 patients of the case group, 63 patients (77.8%) had started HIV treatment, and 18 patients (22.2%) cleared HCV RNA spontaneously **(Table 1).**

**Table 1:** Characteristics of the study population.

	Cases (81)	Controls (243)	*p-value*
Age, median (IQR), years	30.5 (18–52)	28.8 (21–45)	0.54
CD4+, median (IQR) cells/mm3	498 (285–674)	324 (212–499)	<0.001
HIV viral load, median (IQR), copies/ mm^3^	51 (40–47347)	15426 (6321–89363)	<0.001
HCV viral load, median (IQR), copies/mm	94326 (50–514500)		
HCV seroconversion while on ART, n (%)	18 (22.2)		
Receiving ART, n (%)	46 (56.7)	11 (4)	
Genotype, n (%)			
1a, n (%)	50 (79.3)		
1b, n (%)	3 (4.7)		
2b, n (%)	6 (9.5)		
4, n (%)	2 (3.1)		
1a/4, n (%)	2 (3.1)		

Abbreviations: ART: Antiretroviral therapy

### Risk factors

In bivariate analysis we found that cocaine use (OR: 3.36; 95% confidence interval [CI]1.80–6.27; P, = 0.01), sharing straw during cocaine inhalation (OR: 4.28; 95% CI 1.35–13.52; P = 0.01); marijuana use (OR: 2.10; 95% CI 1.24–3.58; P = 0.005); ethyl chloride use (OR: 2.67; 95% CI 1.29–5.56; P = 0.007) and use of poppers (OR: 2.22; CI 1.33–3.7; P = 0.002) were associated with HCV transmission.

Other risk factors associated with HCV transmission were use of sex toys (OR: 1.88; 95% CI; 1.11–3.18; P = 0.018), share sex toys (OR: 6.19; 95% CI 2.33–16.42; P = <0.001), group sex (OR: 1.95; 95% CI 1.11–3.25; P = 0.009), and enemas before sex (OR: 3.2; 95% CI 1.91–5.41: P = <0.001) **(Table 2).**

**Table 2:** Risk factors bivariate analysis.

Risk Factor	OR	95%CI	P Value
Age (>40 years)	3.42	1.75–6.71	<0.001
IV Drugs	2.63	1.05–6.6	0.03
>5 Sex partners	1.03	0.60–1.78	0.89
Tattoos	1.18	0.71–1.98	0.51
Piercing	1.94	1.1–3.2	0.01
Surgery	1.55	0.68–1.95	0.59
Dental surgery	0.82	0.49–1.35	0.44
Colonoscopy	1.87	0.82–4.27	0.13
Acupuncture	0.08	0.40–1.95	0.76
Prision record	0.59	0.06–5.16	0.63
Chemsex	1.52	0.91–2.56	0.17
Cocaine	3.36	1.80–6.27	0.01
Sharing straw for cocaine inhalation	4.28	1.35–13.52	0.01
Use of gamma hydroxybutyrate (GHB)	2.05	0.56–7.46	0.26
Use of ketamine	6.2	1.12–34.84	0.01
Use of ecstasy	1.99	0.06–4.60	0.09
Use of marijuana	2.10	1.24–3.58	0.005
Use of methamphetamine	2.09	1.01–4.32	0.04
Use of poppers	2.22	1.33–3.7	0.002
Use of ethyl chloride	2.67	1.29–5.56	0.007
Alcohol use	1.85	1.04–3.29	0.03
Use of phosphodiesterase 5 inhibitors	1.28	0.67–2.45	0.44
Insertive fisting	1.34	0.53–3.38	0.53
Receptive fisting	1.08	0.44–2.44	0.91
Use of sex toys	1.88	1.11–3.18	0.018
Share Sex Toys	6.19	2.33–16.42	<0.001
Group Sex	1.95	1.11–3.25	0.009
Enemas previous to sex (rectal douching)	3.2	1.91–5.41	<0.001
Syphilis	1.46	0.85–2.50	0.16
Urethritis	1.55	0.84–2.87	0.15
HBV	0.96	0.45–2.06	0.92
HPV	0.85	0.45–1.62	0.63
Ulcerative genital infection	1.30	0.69–2.4	0.40
Proctitis	1.47	0.63–3.40	0.36

Abbreviations: OR: Odds ratio; 95% CI: 95% confidence interval; HBV: Hepatitis B virus and HPV: Human papillomavirus

After entering these variables into a logistic regression model sharing straw during cocaine inhalation (odds ratio [OR]: 9.03; 95% CI; 1.35–13.52; P = 0.003), sharing sex toys (odds ratio [OR]: 17.53, 95% CI 6.85–44.86; P = 0.002), and ethyl chloride use (odds ratio [OR]: 2.26; 95% CI 1.29–5.56; 0.037) remained statistically significant **(Table 3).**

**Table 3:** Multivariate analysis.

Risk factors	aOR	95%CI	P value
• Sharing straw for cocaine inhalation	9.03	1.35–13.52	0.003
• Sharing Sex Toys	17.53	6.85–44.86	0.002
• Ethyl chloride	2.26	1.29–5.563	0.037

Abbreviations: aOR: adjusted odds ratio, 95% CI: 95% confidence interval

## Discussion

In this study, risk factors for the transmission of HCV were assessed in a Mexican HIV-positive MSM population. Independent risk factors for HCV transmission were sharing the straw for inhaling cocaine, as well as sharing sex toys that involve penetration. In addition, the use of ethyl chloride during sexual intercourse was also a risk factor for HCV transmission.

In general, our findings are in agreement with studies from Germany, UK, Japan, France and New York showing that risk factors for transmission of HCV were sex practices involving rectal trauma that leads to bleeding, receptive fisting without the use of gloves, group sex, inhaling cocaine or methamphetamine, receptive anal sex without protection and the use of sex toys [5, 6, 16, 18]; these observations were also supported by a French cohort identifying similar risk factors [19].

In contrast to the studies above, we were not able to find an association between receptive anal sex and HCV infection. Cocaine was only a risk factor when sharing the straw [20]. Using ethyl chloride in chemsex is common in Mexico and may a regional phenomenon and because of this not reported in other studies so far [21].

Sex toys can cause dilation of anal tissue and micro-bleeding during sexual activity and promote HCV transmission by this [22, 23]. It has been suggested that HCV is also transmitted by the toy itself by sharing contaminated sex toys [7, 24]. Indeed, we found an association between sharing sex toys and HCV transmission. However, as case patients may have attributed the use of sex toys with acquiring HCV infection a recall bias compared to uninfected controls cannot be excluded.

In contrast to other studies rectal douching and sexually transmitted infections associated with mucosal ulceration (mainly syphilis) were not associated with transmission of HCV infection in our study [25–33]. This may be partially explained by the fact that we used a VDRL as a surrogate marker for syphilis which cannot discriminate between active ulceration and past infection.

This is the first study carried out in Mexico assessing risk factors for HCV transmission in HIV-positive MSM. Unlike in most other studies, detailed information on a number of risk factors including many substances used in chemsex, as well as other sexually transmitted infections and behavioral factors were assessed in the questionnaire.

Our study has several limitations, mostly by design. It is a case-control study, retrospective analysis performed in a single hospital. Another limitation is that we were not able to pair cases and controls by age or any other characteristic, because HCV/HIV coinfection prevalence in Mexico is low. Correlated variables, e.g., inhaling cocaine and sharing a straw to inhale cocaine or the use of sex toys and sharing sex may affect the precision of the regression model this has to be considered for the result interpretation.

To our knowledge, this is the first study reporting a possible association between ethyl chloride and HCV infection. The reason could be that ethyl chloride is a cheap product and can be easily accessed in drug stores and supermarkets.

In general, the study highlights the necessity to screen HIV-positive MSM for HCV infection. Some of these risk factors could be mitigated by safer practices such as not sharing cocaine straws or sex toys. Assessment of local populations for the main risk factors for HCV transmission may help to develop specifically targeted behavioral interventions to reduce HCV transmission.

## Supporting information

S1 Appendix Risk factors for HCV transmission in HIV-positive men who have sex with men self-questionnaire. (DOCX)
