# Supplementary material for: Risk factors for HCV transmission in HIV-positive men who have sex with men in México
Source: PLoS One. 2022 Jul 15;17(7):e0269977. doi: 10.1371/journal.pone.0269977 (PMC9286238; doi:10.1371/journal.pone.0269977)
Supplement: S1 Appendix — (DOCX) [file pone.0269977.s001.docx]

**Risk factors for HCV transmission in HIV-positive men who have sex with men in México**

All the information provided is STRICTLY CONFIDENTIAL and will only be used for statistical purposes. No results presented in this study will refer to individuals in particular.

Instructions:

Read the following questionnaire carefully, cross out the answer or, if applicable, fill in the corresponding spaces.

Age: _____________ Birthplace: ___________________________ CD4+: ___________________

| **Risk factor** |  | |
| --- | --- | --- |
| 1. Sex while high on drugs | Yes | No |
| 1. Sex while high on cocaine | Yes | No |
| 1. Sex while high on GHB | Yes | No |
| 1. Sex while high on ketamine | Yes | No |
| 1. Sex while high on ecstasy | Yes | No |
| 1. Sex while high on marijuana | Yes | No |
| 1. Sex while high on methamphetamine | Yes | No |
| 1. Sex while drunk | Yes | No |
| 1. Sex while ethyl chloride use | Yes | No |
| 1. Sex while high on poppers | Yes | No |
| 1. Sex while Sildenafil/Tadalafil | Yes | No |
| 1. Sex while high in other substances | Yes | No |
| 1. Receptive anal intercourse | Yes | No |
| 1. Insertive anal intercourse | Yes | No |
| 1. Receptive fisting | Yes | No |
| 1. Insertive fisting | Yes | No |
| 1. Share sex toys | Yes | No |
| 1. Use of sex toys | Yes | No |
| 1. Group sex | Yes | No |
| 1. Previously had syphilis | Yes | No |
| 1. Previously had urethritis | Yes | No |
| 1. Previously had proctitis | Yes | No |
| 1. Previously had ulcerative lesion | Yes | No |
| 1. HBV infection | Yes | No |
| 1. HPV infection | Yes | No |
| 1. Intravenous drugs | Yes | No |
| 1. Having received blood (products) before 1995 | Yes | No |
| 1. Tattoo | Yes | No |
| 1. Body piercing | Yes | No |
| 1. Surgery | Yes | No |
| 1. Surgical/dental procedure | Yes | No |
| 1. Colonoscopy | Yes | No |
| 1. Be in Jail | Yes | No |
| 1. Acupuncture | Yes | No |
| 1. Sharing straw during sniff cocaine | Yes | No |
| 1. Rectal douching | Yes | No |
| 1. HCV-infected mother | Yes | No |
| 1. >5 sexual partners | Yes | No |
| 1. Age >40 years | Yes | No |
| 1. Mother with Hepatitis C |  |  |
| 1. Haemodialysis patient |  |  |
